# Multifaceted Impacts of Periodontal Pathogens in Disorders of the Intestinal Barrier

**DOI:** 10.3389/fimmu.2021.693479

**Published:** 2021-07-27

**Authors:** Yingman Liu, Wenxuan Huang, Jiaqi Wang, Jiaojiao Ma, Manman Zhang, Xiaoying Lu, Jie Liu, Yurong Kou

**Affiliations:** ^1^Department of Periodontics, School and Hospital of Stomatology, China Medical University, Liaoning Provincial Key Laboratory of Oral Diseases, Shenyang, China; ^2^School of Stomatology, Shenyang Medical College, Shenyang, China; ^3^Department of Oral Biology, School and Hospital of Stomatology, China Medical University, Liaoning Provincial Key Laboratory of Oral Diseases, Shenyang, China; ^4^Science Experiment Center, China Medical University, Shenyang, China

**Keywords:** periodontal pathogens, intestinal barrier, gut microbiota, tight junction, immune barrier

## Abstract

Periodontal disease, a common inflammatory disease, is considered a hazardous factor that contributes to the development of diseases of the digestive system as well as other systems. The bridge between periodontitis and systemic diseases is believed to be periodontal pathogens. The intestine, as part of the lower gastrointestinal tract, has a close connection with the oral cavity. Within the intestine, the intestinal barrier acts as a multifunctional system including microbial, mucous, physical and immune barrier. The intestinal barrier forms the body’s first line of defense against external pathogens; its breakdown can lead to pathological changes in the gut and other organs or systems. Reports in the literature have described how oral periodontal pathogens and pathobiont-reactive immune cells can transmigrate to the intestinal mucosa, causing the destruction of intestinal barrier homeostasis. Such findings might lead to novel ideas for investigating the relationship between periodontal disease and other systemic diseases. This review summarizes studies on the effects of periodontal pathogens on the intestinal barrier, which might contribute to understanding the link between periodontitis and gastrointestinal diseases.

## Introduction

As the most important digestive organ and largest immune organ in the human body, the intestine not only absorbs nutrients and water but also maintains the harmonious coexistence of symbionts. To keep such a balance between symbionts, the human intestine has evolved unique regional immune characteristics that have resulted in an intestinal defense barrier known as the “gut barrier” (1). The intestinal barrier is equipped with an intricate multilayer system that is made up of resident gut microbiota, a mucous layer, a monolayer of intestinal epithelial cells, and a complex network of immune cells (2, 3). These components form microbial, chemical, physical, and immune barriers that work both simultaneously and sequentially. Multilayer gut barriers are organized into two main parts: an upper physical part and a lower functional part. The upper physical part, the superficial layer of the intestinal barrier, frustrates bacterial adhesion and regulates the diffusion of materials from the lumen to the underlying tissue. The lower functional part, the deep layer of the intestinal barrier, distinguishes commensal bacteria from pathogens; it is responsible for immune suppression to counter commensals and the immune response to pathogenic microbes and components (4, 5). It has been shown that perturbations to the integrity of the intestinal barrier can aggravate a variety of diseases: for example, arthritis, hepatic disorders, diabetes and inflammatory bowel disease (IBD) (6, 7).

Periodontal disease, a chronic inflammatory disease involving the destruction of gingival tissue and the resorption of alveolar bone, is manipulated by the intricate interaction between the host’s immune system and microbial infection. In the course of the initiation and progression of periodontal disease, a dramatic shift occurs in the subgingival ecology as it changes from a symbiotic to dysfunctional microbial community composed mostly of anaerobic genera. The keystone periodontal pathogens, including *Porphyromonas gingivalis*, *Fusobacterium nucleatum*, and *Aggregatibacter actinomycetemcomitans*, to name a few, are predominant bacterial species of the anaerobic microbiota. The keystone periodontal pathogens are enriched in virulence factors and have adapted to thrive in an inflammatory environment (8, 9). It is recognized that the presence of pathogenic bacteria in periodontal disease correlates with the incidence and progression of several systemic diseases such as cardiovascular disease, diabetes, Alzheimer’s disease, and arthritis (10, 11). Reports in the literature (12–14) have proposed that periodontal pathogens might induce multiple changes in the microbiota community, barrier function, and immune system of the gut, which lead to an increased risk of many systemic diseases associated with low-grade inflammation. Therefore, to explore the multifaceted role of periodontal pathogens in the intestinal barrier might help us understand the mechanism by which periodontitis exerts an impact on systemic diseases.

## The Possible Routes by Which Periodontal Pathogens Influence the Intestine

### The Gastrointestinal Route

The gastrointestinal route is a widely approved route that the oral bacteria travel into intestine. Similar to the gut, the oral cavity is also a major microbial reservoir. The mouth separates internal and external environments and is where bacteria enter the gastrointestinal tract, along with saliva, food, and water (15). The physiological secretion of saliva occurs in the oral cavity at the rate of 0.75~1.5L per day from mainly three pairs of salivary glands (16). Saliva is sterile when excreted into the mouth; however, saliva becomes contaminated with a conglomerate of bacteria after they are shed from the oral surface (17, 18). Under physiological conditions, the salivary flora rarely arrived to the gut because of the protection from the gastric acid and alkaline bile. However, the salivary flora of patients with periodontitis is remarkably distinct from that of orally healthy controls. For example, the salivary abundance of *P. gingivalis* was significantly higher in patients with periodontal disease (19, 20). According to a quantitative analysis, it was estimated that patients with severe periodontitis swallowed approximately 10^12^~10^13^ *P. gingivalis* bacteria per day (21–23). Because of it is acid resistant, *P. gingivalis* might pass through the stomach and reach the intestinal tract, destroying intestinal homeostasis (24). This hypothesis was further confirmed by the discovery of *P. gingivalis* in the intestinal tract. Arimatsu and his colleagues ferreted out *P. gingivalis* in the jejunum and ileum of *P. gingivalis*-treated mice one hour after oral administration; its proportion in the microbial flora was diminished by 3 hours. At 16 hours, *P. gingivalis* was discovered in the colon (25). Another study also described how *P. gingivalis* was detected in both fecal and cecal samples (26).

Of course the majority of oral bacteria are eliminated by the gastric acid and alkaline bile. However, the impairment of the physiological protection can allow microbiota translocation and communication. For example, the gut colonization by oral bacteria is significantly augmented in patients who have achlorhydria-related gastric dysfunction. Besides, the transmission probability of oral bacteria, including periodontal pathogens, from oral cavity to intestine, is increased in patients with systemic diseases such as colorectal cancer and IBD (13, 27).

### Blood Route

The hematogenous route is one of the important routes for oral bacteria dissemination to other organs. The extremely well vascularization and gingival ulceration in periodontal pockets in the connective tissues of the periodontium allow the periodontal pathogens to readily enter the bloodstream (9, 28). Plaque accumulation and gingival inflammation significantly increase the prevalence of bacteraemia. The theory also supported by the studies that periodontal pathogens such as *P. gingivalis* and *F. nucleatum* were detected in the blood of experimental periodontitis models and traveled to other organs such as liver, spleen and vascular samples (29, 30). A study proved that the hematogenous route appeared much more resultful than the digestive tract for fusobacteria trafficking to colorectal cancer (CRC) (31). It was biologically more plausible that oral fusobacteria spread to CRC through hematogenous route, because *Fusobacteria* was absent from biofilms in the colonic mucous layer of adenomas of familial adenomatous polyposis patients and some sporadic CRC patients (32).

### Immune Cell Migration Route

Given that oral and colonic mucosae are physically correlative, it is undoubtable that oral bacteria transmigrate to the lower gastrointestinal tract and presumably trigger pathogenic immune responses (33). John Bienenstock presented a concept of “common mucosal immune system” and speculated that the mucosal immune system might be a system-wide “organ” in which the immune cells distributed throughout the body could interplay among different mucosal tissues (34). In addition, it has been shown that immune cells derived from oral draining lymph nodes are able to migrate to other lymphoid tissues, including but not limited to the gut (35). Consequently, it was likely that periodontal inflammation given rise to the advent of periodontal pathobiont-reactive T cells in the lymph nodes draining the oral cavity. The pathobiont-reactive T cells were then incited by periodontal pathogens to migrate from the oral mucosa to the gut where they activated intestinal immune cells and caused intestinal inflammation (36).

**Figure 1:**
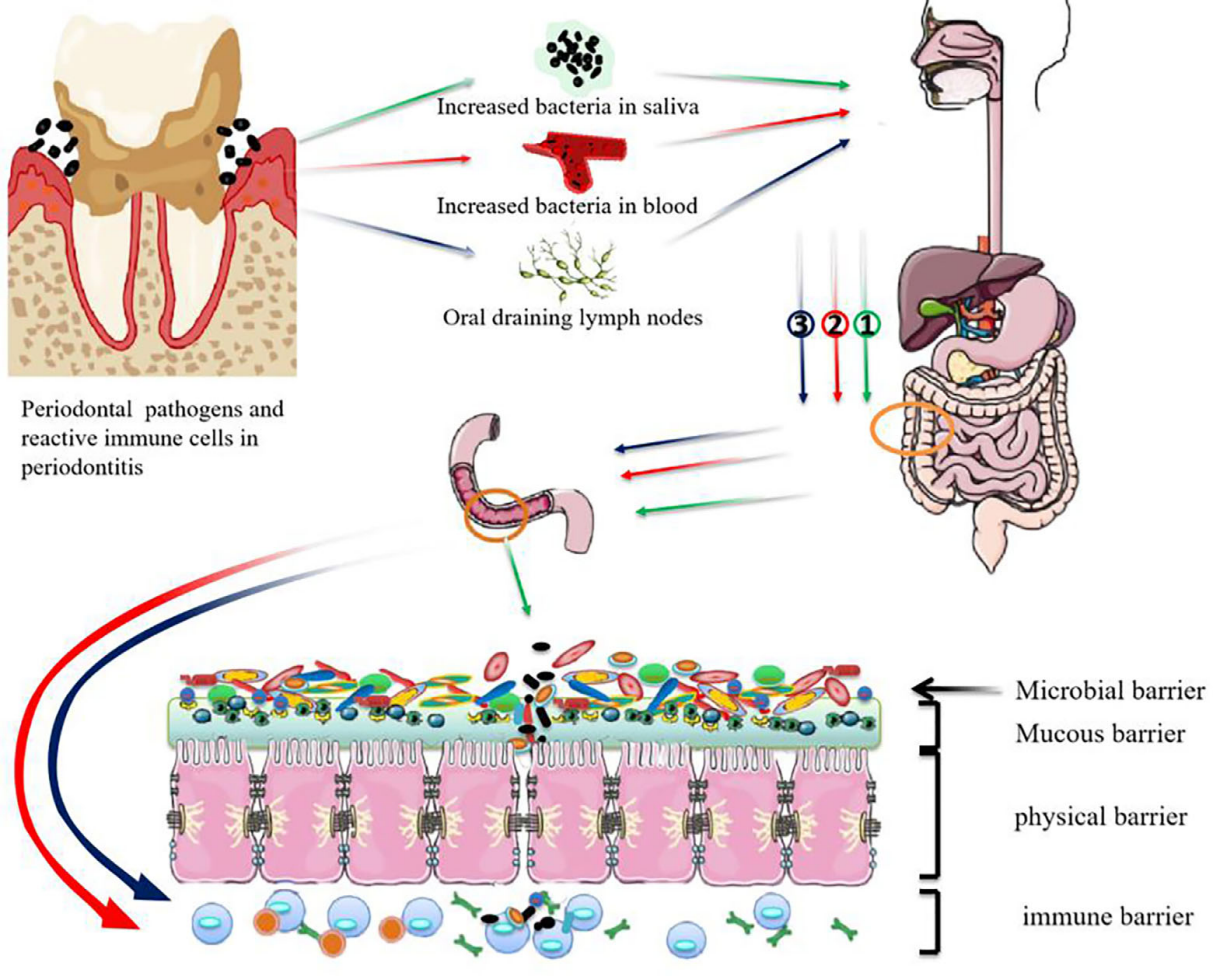
Possible mechanisms for periodontal pathogens damaging the intestinal barrier. ① Swallowed periodontal pathogens may transmigrate to the lower gastrointestinal tract and disrupt intestinal homeostasis. ② periodontal pathogens may disseminate to intestinal tissues through the bloodstream and take part in the progression of intestinal diseases. ③ Pathobiont-reactive immune cells activated by periodontal pathogens may transmigrate from the oral draining lymph nodes to the gut where they activate intestinal immune cells.

Both bacterial ectopic colonization and lymph node drainage of immune cells have been recognized as the ways by which periodontal pathogens disrupt intestinal barrier homeostasis (**Figure 1**). However, we still don’t know whether periodontal bacteria travel to the gut as free bacteria or by means of erythrocytes or lymphocytes. It is also unclear how the periodontal pathogens travelling to the gastrointestinal tract can survive the gastric acid and alkaline bile. The mechanism that regulates the immune cell migration between oral and colonic mucosae is also to be illuminated.

## Effects of Periodontal Pathogens on Intestinal Microbial Barrier

### Composition and Function of Intestinal Microbiota

Microbiota denotes the entire population of microorganisms that colonizes a specific region, including not just bacteria but also other microbes such as fungi, protozoans, viruses, and archaea (37, 38). The majority of microorganisms residing in humans are found in the gut, forming the largest microbial ecosystem of the body. Culture-based studies have led to the proposal for a core microbiota since nearly all healthy grown-ups have been found to consistently share the majority of gut bacterial species (39). The four core bacterial phyla are present in the adult human gut: Bacteroidetes and Firmicutes are the most predominant, followed distantly by Actinobacteria and Proteobacteria (40). Despite the uniformity of these main components of the intestinal microbiota, their relative proportions, as well as the other species present, vary strikingly across individuals. Over a person’s lifespan, subtle temporal and spatial variations occur in the distribution of intestinal microorganisms (41). It is generally believed that the development of the microbiota begins from birth (42, 43) and only reaches a relatively distinct, stable microbiota community by about two or three years of age (44). Spatially, the distribution of the gut microbiota is stratified along not only a longitudinal but also transverse axis. In longitudinal axis, from the small intestine to the colon, facultative anaerobes are thought to survive in the small intestine while a dense and diverse community of anaerobes has been shown to dominate the colon. In the transverse axis, from the mucous layer to lumen, the composition of the microbiota is significantly dissimilar in the mucous layer and feces/lumen. For example, Bacteroidetes appears to be more abundant in fecal/luminal samples than in the mucus. Compared with the gut lumen, Firmicutes, specifically *Clostridium* cluster XIVa, are mainly enriched in the mucous layer.

As an involved ecological community, the microbiota in our gut assists us to stay healthy by exerting essential multivarious functions. Presently, the intestinal microbiota exhibits three major functions (45, 46): (i) metabolic activities with respect to salvaging energy and absorbing nutrients; (ii) trophic effects on intestinal epithelial cells, and the immune system and function; and (iii) shield of the colonized hosts against the attack by alien microorganisms. The co-evolution of the host and microbes contributes to many host physiological processes. However, impaired regulation in the microbiota may generate diseases such as intestinal inflammation and cancer. It has been clearly shown in animal experiments that the microbiota functions in the pathogenesis of IBD and intestinal neoplasia (47, 48). In support of this, in the absence of the commensal microbiota under gnotobiotic conditions, experimental animals did not develop colitis though they did in conventional environments (49). Additionally, the carcinogenic agent, azoxymethane, induced colon tumors in colitis-susceptible interleukin (IL)-10 knockout mice colonized with certain commensal bacteria but failed to do so in gnotobiotic IL-10 knockout mice (50). The duration and severity of chronic colitis are the significant risk factors for colitis-associated colon cancer (CAC). The IL-10 knockout mice develop spontaneously colitis on account of the intolerance to intestinal bacteria and microbiota-induced activation of effector T cells. Thus the gnotobiotic IL-10 knockout mice failed to develop the colitis and subsequent CAC. Besides, the intestinal bacteria such as *Bacteroides fragilis*, *E. coli*, and so on, promoted the proliferation of tumor cells and the development of colon cancer *via* activating diverse signaling routes (51, 52).

### Effect of Periodontal Pathogens on Intestinal Microbiota

As periodontal pathogens translocate to the intestine, the concomitant variation in gut microbiota was affirmed. Firmicutes was reduced and Bacteroides increased at the phylum level after *P. gingivalis* was given to C57BL/6N mice. Because *P. gingivalis* was not detected in the blood and the proportion of *P. gingivalis* in the gut was very low, systemic inflammation might have occurred because of variation in the gut microbiota induced by *P. gingivalis*. Sasaki et al. found that the composition of the intestinal microbiota was also altered by intravenous injection of inactivated *P. gingivalis* in mice, although sonicated *P. gingivalis* did not directly reach the gut (53). Changes included that the phyla Tenericutes and Proteobacteria tended to decrease. Unsurprisingly, findings in a clinical case-control study were consistent with those in animal experiments. Lourenco et al. compared the intestinal flora of individuals with healthy periodontium, gingivitis, or periodontitis. It was found that the variation in intestinal flora was significant, with decreased microbial diversity and a high Firmicutes/Bacteroides ratio associated with disease severity. In addition, periodontally healthy and diseased groups could be distinguished based on the composition of the microbiome in stool samples. The identification of the gut microbiota, especially the *Mogibacteriaceae* and *Ruminococcaceae* families and the genus *Prevotella*, might be able to aid in the classification of diseased patients (54).

The alteration in intestinal flora caused by periodontal disease is not entirely consistent. In terms of the two major phyla in human intestinal flora, a tendency for decreased Bacteroides and increased Firmicutes in a *P. gingivalis-*administered mouse group in comparison to a control mouse group was shown (12). However, a contrary finding has been described in the literature: that is, a high Bacteroides/Firmicutes ratio was reported (26, 55). Ohtsu administered *P. gingivalis* to streptozotocin-induced diabetic mice and found no noticeable change in the abundance of intestinal Bacteroides (56). Contradictory tendencies for Firmicutes and Bacteroides have also been reported in several systemic inflammatory conditions, such as rheumatoid arthritis (RA) (57, 58) and obesity (59, 60) as well as others. Hence, confounding factors, including diet, obesity, and sampling sites, have been involved in alterations of intestinal flora caused by periodontal pathogens.

The gut microbiota can be disturbed by the massive long-term oral ingestion of periodontal pathogens, which is deemed a potential mechanism that links periodontitis to inflammatory systemic diseases. In a study by Komazaki et al., C57BL/6J mice orally administered *Aggregatibacter actinomycetemcomitans* for 12 weeks presented with impaired glucose tolerance, insulin resistance, and a perturbation of the gut flora (61). Specifically, in *A. actinomycetemcomitans*–administered mice, the genus *Turicibacter* was underrepresented. This genus reportedly produced butyric acid, which improved insulin sensitivity. Coincidentally, in C57BL/6J mice fed a high-fat diet, recipients of oral *P. gingivalis* showed inhibited glucose uptake in skeletal muscles, with higher tumor necrosis factor (TNF)-α expression and lower insulin signaling. At the same time, *Turicibacter* was underrepresented in the gut of mice treated with oral *P. gingivalis*. These findings suggest that the administration of periodontal bacteria may negatively affect insulin resistance by decreasing the abundance of *Turicibacter* in the gut, thereby decreasing insulin sensitivity.

In addition to metabolic disorders, it is also considered that the “oral-gut” axis was connected to neuroinflammation. According to epidemiological studies, periodontitis was recognized as a risk factor for cognitive impairment. Scholars speculated that chronic periodontitis evoked a neuroinflammatory response, as well as subsequent neurodegeneration and cognitive decline because of a disorder in a “microbiota–gut–brain” axis (62). Moreover, in mice with chronic periodontitis, alteration of the microbiota at the phylum and genus levels was significantly found in saliva and feces. Meanwhile, intestinal and blood–brain barriers were destroyed, and endotoxin and pro-inflammatory cytokines at the mRNA level were upregulated systemically and in the brain. One study detected a systemic inflammatory response after changes in the intestinal flora in *P. gingivalis-*administered mice, which supported the idea that the perturbation of intestinal microbiota by periodontal pathogens might be a mechanism connecting periodontal disease and systemic diseases (26). These findings suggested indirectly that periodontal pathogens might travel to the intestinal tract and alter the composition of the intestinal flora (**Table 1**).

**Table 1:** Main studies about the effects of periodontal pathogens on the intestinal microbiota.

Objects of study	Comparison^1^	Phylum	Class	Order	Family	Genus
DBA/1J mice (24)	*P. gingivalis*-vs sham- and *P. intermedia*-	Bacteroidetes ↓				Bacteroidetes ↓
		Firmicutes↑				Prevotella ↓
						Allobacullum↑
C57BL/6N mice (25)	*P. gingivalis*- vs sham-	Bacteroidetes↑				
		Firmicutes↓				
C57BL/6 mice (26)	*P. gingivalis-* vs sham-administered mice	Bacteroidetes↑				S24-7 and Prevotella↑
		Firmicutes↓				unclassified Clostridiales↓
C57BL/6J mice fed high-fat diet (53)	*P. gingivalis*- vs control	Tenericutes↓			Alcaligenaceae↑	Bilophila ↓
		Proteobacteria↓			Erysipelotrichaceae↑	Dehalobacterium ↓
					Dehalobacteriaceae ↓	Sutterella ↑
					Ruminococcaceae↓	Allobaculum↑
						Faecalibaculum rodentium↑
						Lactobacillus johnsonii↑
						Lactobacillus reuteri ↑
Patients (54)	Chronic periodontits vs gingivitis vs healthy patients	Firmicutes↑		Lactobacillales(gingivitis)^2^↑	Comamonadaceae(gingivitis)^2^↑	Prevotell(gingivitis)^2^↑
		Proteobacteria↑			Mogibacteriaceae↑	Selenomonas noxia↑
		Verrucomicrobi↑			Ruminococcaceae↑	Leptotrichia↑
		Euryarchaeota↑				Tannerella↑
		Bacteroidetes ↓				Campylobacter↑
Male ApoE^-/-^ mice (55)	*P. gingivalis-* vs control	Bacteroidetes↑	Bacilli↓		S24-7↑	Anaeroplasma ↑
		Firmicutes↓	Clostridia↓		Lachnospiraceae↓	
		Tenericutes↑			Ruminococcaceae↓	
					Anaeroplasmataceae↑	
					Erysipelotrichaceae↑	
Mice with diabetes (56)	*P. gingivalis*- vs control	Deferribacteres↑				Lactobacillus↑
						Turicibacter↓
						Mucispirillum↑
Wild-type mice (56)	*P. gingivalis*- vs control	Deferribacteres↑				Lactobacillus↓
						Turicibacter↑
						Mucispirillum↑
C57BL/6J mice fed normal chow (61)	** ***A. actinomycetemcomitans-* vs control					Turicibacter↓
C57BL/6J mice fed high-fat diet (61)	*A. actinomycetemcomitans-* vs control					Turicibacter↓
C57BL/6J mice (62)	Chronic periodontitis- vs control	Firmicutes↑				Dubosiella↑
		Verrucomicrobia↑				Muribaculum↑
		Acidobacteria↑				Butyricicoccus↑
		Tenericutes ↓				Lactobacillus↓
						Delftia↓
						Anaeroplasma↓
						Gordonibacter ↓
C57BL/6 mice (12)	*P. gingivalis*- vs control	Bacteroidetes ↓			S24-7↓	unclassified Coriobacteriaceae↑
		Deferribacteres↑			Paraprevotellaceae↓	Gemellaceae↑
					Mogibacteriaceae↓	Clostridiaceae↑
					Deferribacteriaceae↑	unclassified S24-7↓
					Gemellaceae↑	Prevotellaceae↓
					Clostridiaceae↑	Mogibacteriaceae↓
						Dorea↓
						Butyricicoccus↓
						Bilophila↓

^1^Comparison condition A vs condition B: ↑ Increase in condition B relative to condition A, ↓ Decrease in condition B relative to condition A, ns not significant.

^2^The changes are the most significant in gingivitis patients.

## Effect of Periodontal Pathogens on Intestinal Mucous Barrier

The mucous barrier, an often ignored part of the intestinal barrier system, is the first physical defense that separates bacteria in the lumen from the epithelium. The main building blocks of the mucous barrier are highly glycosylated mucin proteins that form a gel-like sieve structure overlying the intestinal epithelium. The mucin 2 (MUC2) secreted by goblet cells is the most abundant mucin protein in small and large intestine (63). The small intestine only has one mucous layer, whereas the colon has two layers: a loose outer layer and a dense inner layer. The loose outer layer allows the colonization of indigenous or transient microorganisms. The dense inner, devoid of bacteria, is firmly adherent to the epithelium (64–66). The mucous components of the gut barrier are fortified by glycoproteins, antimicrobial peptides (AMPs) and proteins including lysozyme. These AMPs including alpha-defensins, lysozyme and Reg3 proteins are secreted by Paneth cells and enterocytes to protect the epithelium from a variety of insults (67, 68). Thus, the mucous layer is also a chemical barrier (69). Secretory IgA (SIgA) molecules represent another important constituent of the intestinal mucous surface. IgA is produced by local plasma cells in the mucosal lamina propria and then transported into gut secretions by the polymeric immunoglobulin receptor (pIgR). In secretions, the extracellular domain of pIgR released by proteolytic cleavage covalently bounds to IgA to form the SIgA complex. MUC2 and pIgR act coordinately to localize SIgA in the outer mucous layer (70). In the outer mucous layer, SIgA binds bacteria-derived and food-derived antigens to retain toxins or bacteria within the lumen (67).

If mucin production or processing is defective, the mucous layer will fail to restrict bacterial access to the epithelia. MUC2 is important in maintaining the integrity of intestinal barrier, as MUC2 knock-out mice spontaneously developed inflammation and colon cancer (71–73). Gingipains are a group of complex arginine- or lysine-specific cysteine proteinases (also widely known as RgpA, RgpB, and Kgp) that have been acknowledged as one of the essential virulence factors of *P. gingivalis*. They are potent “trypsin-like” proteinases, capable of cleaving or degrading a series of host proteins including cytokines, extracellular matrix proteins, plasma proteins and host cell surface proteins (74). A study demonstrated that RgpB secreted by *P. gingivalis* was able to cleave MUC2 at a specific site where it would disrupt the MUC2 polymeric network (75). Animal study findings have revealed that AMPs are crucial in the prevention and clearance of intestinal pathogens (76–78). However, *P. gingivalis* is able to circumvent and even manipulate AMPs, which has been demonstrated in periodontal disease and atherosclerosis (79). These detrimental effects of *P. gingivalis* on mucous layer may contribute to the further disruption of intestinal homeostasis.

## Effect of Periodontal Pathogens on the Intestinal Physical Barrier

### Composition and Function of the Intestinal Physical Barrier

Underneath the mucous layer is the intestinal physical barrier. It is mainly made up of intestinal epithelial cells and tight junctions (TJs) by which epithelial cells are linked together. The integrity and regenerative capacity of the intestinal epithelium are known to be the structural basis of the intestinal physical barrier. The specialized epithelium is composed of a monolayer of columnar cells that forms the largest barrier that connects the internal tract with a changing external environment (80). TJs play several vital roles in the intestinal barrier. By controlling intestinal epithelial permeability, TJs are major players in the functions of the intestinal physical barrier. They are located at the most apical–lateral position between adjacent epithelial cells and consist of multiple proteins, including occludin, claudins, zona occludens (ZOs), and adhesion molecules. Occludin is essential for the maintenance of the homeostasis of the intestinal epithelial barrier (81). The decreased expression of occludin means an increase in gut permeability (82). At present, at least 27 different claudin proteins have been detected, forming a skeleton structure of TJs. Mounting evidence has highlighted how different claudin proteins play different roles in the intestinal barrier. For example, claudins -1, -3, -4, -7, and -8 reduce the transport of solutes and water, enhancing the barrier effect, while claudins -2, -10, -12, and -15 do the opposite (83). The ZO family, including ZO-1, ZO-2, and ZO-3, is the most studied. ZO proteins act as scaffolds by connecting with other transmembrane proteins (84).

An intestinal physical barrier that regulates the flow of nutrients, ions and water between the lumen and underlying tissues restricts contact between the host and intraluminal exogenous antigens and microbes (85). The destruction of an intestinal physical barrier and an increase in intestinal epithelial permeability are common pathophysiological mechanisms in diverse diseases (84, 86, 87), such as Parkinson’s disease (88), liver cirrhosis (89), and IBD (90), among others. These diseases present with common alterations in the content and distribution of proteins in the physical barrier. If the content or distribution of the proteins is maladjusted, three effects might ensue: (i) a damaged paracellular pathway; (ii) enhanced paracellular transport of solutes and water; and (iii) increased permeability to macromolecules (91). A disruption of the physical barrier structure can be both a cause and an effect of disease (92).

### Effect of Periodontal Pathogens on the Intestinal Physical Barrier

The intestinal physical barrier can be regulated by a variety of factors. Research on the effect of periodontal pathogens on the intestinal physical barrier has mainly focused on the destruction of TJ proteins induced by *P. gingivalis*. The lipopolysaccharide (LPS) from *P. gingivalis*, known as endotoxin, elicits a broad spectrum of biological responses. In the gut, LPS has been identified as a major irritant that causes intestinal barrier dysfunction. Studies have shown that an instant increase in the permeability of the small intestine for 3 hours in mice could be induced by a high-dose intravenous LPS injection (93); this was most pronounced in the ileum (94). Nakajima et al. found that tight junction protein-1 (ZO-1) and occludin were downregulated at the mRNA level after a single oral administration of *P. gingivalis*, with elevated endotoxin found in the serum (26).

What’s more, Tsuzuno and co-workers concluded that the expression of ZO-1 in the intestine of mice treated with *P. gingivalis* was dramatically decreased at the protein rather than mRNA level (95). The possible involvement of gingipains in the degradation of intestinal ZO-1 protein was suggested by the use of gingipain-deficient *P. gingivalis*. Based on the ability of *P. gingivalis* to invade epithelial cells, a possible mechanism whereby the bacterium might penetrate mucus and intestinal epithelial cells, then degrade ZO-1 using gingipains was proposed (96).

In addition, TJ components are regulated by proinflammatory cytokines driven by the oral administration of *P. gingivalis* (97, 98). Oral *P. gingivalis* induced serum IL-17A, with increased IL-1β and TNF-α found in the colon, and decreased the TJ protein, ZO-1, in R1441G mice (99). *P. gingivalis*–induced IL-17A was less likely to originate from intestinal peripheral lymphoid tissues since IL-17A was not found in the colon. Oral *P. gingivalis* was found to impair intestinal epithelium permeability and induced an augmentation of peripheral IL-17A. Downregulated TJs and upregulated proinflammatory cytokines were observed in the ileum of mice orally administered *P. gingivalis* in a research study by Arimatsu (25). *P. gingivalis* was not found in the blood, so the increased endotoxins might have been derived from intestinal flora because of the destroyed barrier in the intestinal epithelium. Flak and colleagues (100) verified the hypothesis that *P. gingivalis* could aggravate joint inflammation by the dysregulation of gut barrier function. They found that in mice with inflammatory arthritis, the inoculation of *P. gingivalis* raised the endotoxin concentration and the whole bacterial load of the inner mucous layer and lamina propria of the colon, which were presumably related to a downregulation of the TJ molecule, ZO-1.

Meilian et al. declared that the oral administration of *P. gingivalis* was insufficient to represent the effects of periodontitis because of the failure to initiate periodontal lesions. Thus, they evaluated the gut mechanical barrier in mice with experimental periodontitis induced by *P. gingivalis*–adhered tooth ligatures. In accordance with the published literature, expression levels of TJ proteins changed in the ileum and the epithelial barrier became damaged in mice with experimental periodontitis (101).

From the above, it can be concluded that virulent factors from *P. gingivalis* and the intestinal inflammation the bacterium causes might induce enhanced intestinal permeability in the gut epithelium (**Table 2**).

**Table 2:** Main studies about the effects of periodontal pathogens on the intestinal physical barrier.

Objects of study	Comparison^1^	Changes in Physical barrier	Methods
human colonic epithelia and CHO K1 Cells (75)		MUC2↓	Western blot
C57BL/6 mice (26)	*P.gingivalis-* vs control	OCLN and Tjp1↓	qRT-PCR
mice with colitis (95)	*P.gingivalis-* vs sham-	ZO-1↓ E-cadherin(ns)	Western blot
Caco-2 cells (95)		ZO-1↓ E-cadherin(ns)	Western blot
LRRK2 R1441G mice (99)	*P.gingivalis-* vs control	ZO-1↓	Western blot
Mice (25)	*P.gingivalis-* vs control	Tjp1↓	PCR
arthritic mice (100)	*P.gingivalis-* vs control	Tjp1 and E-cadherin↓	qRT-PCR and Immunofluorescence staining
C57BL/6J mice (101)	Periodontitis vs control groups	Occludin and claudin2↑	immunohistochemistry
NCM460 cell, FHC cell (102)		ZO-1 and occludin↓	Western blot
mice (102)	*F. nucleatum-* vs PBS-	ZO-1 and MUC2 ↓	Immunohistochemistry
mice with colitis (102)	*F. nucleatum* + DSS- vs DSS-	ZO-1 and occludin ↓	Western blot

^1^Comparison condition A vs condition B: ↑ Increase in condition B relative to condition A, ↓ Decrease in condition B relative to condition A, ns not significant.

## Effect of Periodontal Pathogens on the Intestinal Immune Barrier

### Composition and Function of the Intestinal Immune Barrier

The surface of the human gastrointestinal tract is estimated to be about 200–400 m^2^, forming the largest surface for interplay between humans and the external environment. The intestinal tract, as one of the largest lymphoid organs, possesses up to 70% of the body’s total immunocytes (103). The immune barrier in intestine is formed by innate and adaptive immunity. In the epithelium and lamina propria, the initiation of the innate immune response is triggered by recognition of pathogen-associated molecular patterns (PAMPs) by pathogen-recognition receptors (PRRs) (e.g., toll-like receptors (TLRs), nucleotide-binding oligomerization domain proteins, retinoic-acid-inducible gene-I, C-type lectins) (104). The PRRs, localized either within endosomes or in the cell surface, can be expressed by intestinal epithelial cells, stromal cells, dendritic cells, macrophages and B and T cells (105). The cells of the intestinal adaptive immune system are distributed regularly throughout the mucosa in the form of organized lymphoid tissue structures of gut-associated lymphoid tissue (GALT), or lamina propria, with intraepithelial cell populations (106). Such intestinal immune tissue can principally be grouped into inductive and effector sites. Priming adaptive immune cell responses in the intestine are located principally in the organized lymphoid structures of GALT and draining lymph nodes. Quantities of effector immune cells pervade the lamina propria and overlying epithelium (107). Particulate antigens are taken and transported by inductive immune cells from the lumen into an underlying dendritic cell (DC)-rich domain, where particulate antigens can be presented to effector immune cells (108).

The functions of the intestinal immune barrier are two-fold: (i) the rapid recognition of antigens, such as foreign microorganisms and toxins, and the induction of an immune response; and (ii) an immune tolerance to nutrients or harmless antigens such as probiotics. The intestinal immune system must orchestrate a complex balance between immune activation *versus* tolerance to maintain intestinal homeostasis. A breach of this balance can result in the occurrence of intestinal (109) and parenteral diseases (110).

### Effect of Periodontal Pathogens on the Intestinal Immune Barrier

#### Effect of *F. nucleatum* on Intestinal Immune Barrier

*F. nucleatum*, identified as a normal commensal bacterium that generally exists on tooth surfaces of healthy individuals, bridges different bacterial species to form tooth plaque (111). *F. nucleatum*, as an opportunistic pathogen, can ectopically colonize extra-oral sites under unhealthy conditions of the body and is associated with a wide range of human diseases. Virulence-related factors from *F. nucleatum* stimulate the progression of intestinal cancer and negatively influence therapeutic efficacy through interactions with the local immune system (112). In support of this, a high concentration of *F. nucleatum* was found in colorectal carcinoma tissues (113). Moreover, the intestinal *F. nucleatum* of patients with colorectal cancer possibly originates from the oral cavity (114). *F. nucleatum* engages with intestinal tumorigenesis by a number of mechanisms. *F. nucleatum* binds to tumor cells and protects them from the cytotoxicity of natural killer cells *via* an interaction between the *Fusobacterial* Fap2 protein and the T-cell immunoglobulin and ITIM domain (TIGIT) receptor expressed on all natural killer cells in humans (115). In mice fed *F. nucleatum* and having tumors, myeloid-derived immune cells that can impede T-cell proliferation and evoke T-cell apoptosis were expanded by the bacterium (116, 117). Herein, *F. nucleatum* results in the immune escape of tumor cells by modulating the tumor immune microenvironment. *F. nucleatum* also can target tumor cell itself and activate TLR4/MYD88/NF-κB, E-cadherin/β-catenin and other pathways to promote the proliferation of colorectal cancer cells (118, 119). In the microenvironment of cancers, *F. nucleatum* infection increases M2 macrophage polarization through a TLR4-dependent mechanism to enhance tumor growth (120). In contrary, a study showed that Autoinducer-2 of *F. nucleatum* promoted microphage migration and M1 polarization (121). Macrophages polarize to distinct functional phenotypes: an M1- or M2-phenotype according to the microenvironment. M1-type polarization is pro-inflammatory, releasing inducible nitric oxide synthase, TNF-α, and IL-8. M2-type polarization is anti-inflammatory, and tumor-promoting, releasing IL-4, IL-10 and transforming growth factor (TGF)-β (121, 122). The contradictory results may be due to a variety of virulent factors of *F. nucleatum* contributing to the development of cancer by different mechanisms (121). Furthermore, several studies (102, 123, 124) demonstrated that *F. nucleatum* also facilitated the intestinal inflammatory disease *via* acting on the gut immunity.

#### Effect of *P. gingivalis* on Intestinal Immune Barrier

In addition to *F. nucleatum*, an increasing number of studies have shown that *P. gingivalis* initiates the inflammatory response in both the periodontium and other tissues (9). A histopathological analysis indicated that intestinal inflammation was identified by an increased infiltration of macrophages, neutrophils, lymphocytes, and plasma cells in the lamina propria of the gastrointestinal tract in mice with experimental periodontal disease (125). In a study on the relationship between periodontitis and systemic inflammation, Palioto et al. developed experimental models of periodontitis in C57BL/6 mice by oral gavage, ligature, or a combination of oral gavage and ligature. It was demonstrated that the expression levels of inflammatory and epigenetic markers were elevated, which included interleukin-18 receptor 1 (IL-18R1, inflammatory marker), DNA methyltransferases 3b (DNMT3b, *de novo* methylation marker), and B and T lymphocyte attenuator (BTLA) in the gut and serum of mice with experimental periodontitis (126). Periodontitis is recognized as an environmental trigger of RA in which *P. gingivalis* may be involved in its pathogenesis (127). Most RA cases are associated with the activation of bacterial citrullination and anti-cyclic citrullinated peptide antibodies. Therefore, *P. gingivalis*, as the only known periodontal pathogen synthesizing peptidylarginine deiminase (PPAD) that is capable of inducing the citrullination of fibrinogen and vimentin, has attracted much attention (128). However, during inflammatory arthritis, PPAD deletion in *P. gingivalis* mainly exacerbated *P. gingivalis*–induced joint inflammation. Therefore, it is questioned whether PPAD synthesized by *P. gingivalis* influences the progression of RA (129–131). A hypothesis was proposed that immune responses in the gut that were elicited with *P. gingivalis* inoculation exacerbated RA (132). T helper 17 (Th17) cells have been proven to be involved in the progression of RA by regulating the adaptive immune response, particularly at mucosal surfaces in the intestine (133). Periodontal pathogens skew the type II collagen–characteristic T-cell immune response in lymph tissues drawing arthritic joints toward the Th17 phenotype (134). And in a study (24) where *P. gingivalis* was orally administered to mice with RA, the proportion of Th17 cells was significantly elevated in mesenteric lymphocytes and Peyer’s patches. Therefore, the association of periodontal disease with RA might be due to effects on intestinal immune responses exerted by ingested *P. gingivalis*.

Clinical studies revealed that *Porphyromonas* was enriched in the stool samples and colorectal mucosa of colorectal cancer patients (135, 136). *P. gingivalis*, as the member of *Porphyromonas*, might be associated with the colorectal cancer. In *vitro*, *P. gingivalis* changed the tumor immune environment by selectively expanding myeloid-derived immune cells and induced CRC development by activating hematopoietic nucleotide-binding oligomerization domain receptor and pyrin domain containing 3 (NLRP3) (137).

Collectively, periodontal pathogens can induce or inhibit the intestinal immune response *via* a variety of pathways. A breakdown in the integrity of the gut immune barrier exerts a threat to normal physiological functions in humans (**Table 3**).

**Table 3:** Main studies about the effects of periodontal pathogens on the intestinal immune barrier.

Periodontal pathogens	Objects of study	effector	Target cells	receptor	Related signals	Related function
*F. nucleatum*	Primary Human NK Cells	Fap2	Nature killer cells	TIGIT		The inhibition of cytotoxicity (115)
	Patients		myeloid-derived immune cells			The augmentation of myeloid-derived immune cells (117)
	CRC cell lines, C57BL mice		CRC cells	TLR4	TLR4/Myd88/NF-κB/miR21	The proliferation of CRC cells (118)
	Human colon cancer cells HCT116, DLD1, SW480, and HT29	FadA	CRC cells	E-cadherin	E-cadherin/β-catenin	The proliferation of CRC cells (119)
			Tumor associated macrophages	TLR4	TLR4/IL-6/p-STAT3/c-MYC	macrophage M2 polarization (120)
	U937 cells	AI-2	macrophage	TNSF9	TNFSF9/IL-1β	macrophage M1 polarization (121)
	Clinical samples, mice, intestinal epithelial cells		epithelial cells	NOD2	NOD2/CARD3/IL-17F/NF-κB	Inflammation (123)
	HT29 cells, C57BL/6 mice	OMV	epithelial cells	TLR4	TLR4/ERK/CREB/NF-κB	Inflammation (124)
	Jurkat cells	Fap2, RadD	T, mononuclear and polymorphonuclear cells		NF-κB, ICE	The induction of apoptosis (138)
*P. gingivalis*	C57BL/6 mice		B, T cells	DNMT3b, BTLA		Inflammation (126)
	DBA/1J mice		Th17 cells			Inflammation (24)
	Human specimen, C57BL/6J mice		myeloid-derived immune cells			The augmentation of myeloid-derived immune cells (137)
			hematopoietic cells	NLRP3		colorectal tumor growth (137)
	C57BL/6 mice		macrophage			macrophage M1 polarization (139)
*P. nigrescens*	C57BL/6 mice		macrophage			increased the M1/M2 macrophage ratio (139)

## Conclusions

In the gut, microbial, immune, and physical barriers interact with each other. For example, the intestinal flora is crucial to the development of host immunity; in turn, the immune system regulates the intestinal flora by immune tolerance and immune rejection (140, 141). The ectopic colonization of periodontal pathogens can cause an intestinal micro-ecological imbalance and inflammation. Meanwhile, virulence factors from periodontal pathogens can destroy the physical intestinal epithelial barrier, allowing bacteria and metabolites of the intestinal lumen to leak into the bloodstream. In addition, periodontal pathogens directly or indirectly regulate the immune system and destroy homeostasis (125, 139).

Recent studies have indicated that the periodontal pathogens, pathogenic to the intestinal barrier, are associated with the progression of a variety of intestinal diseases. However, information on the mechanisms by which periodontal pathogens disrupt the intestinal epithelial barrier is still far from sufficient. Because many diseases pertain to a compromised function of intestinal barrier (81, 142, 143), future studies that dissect the specific mechanisms of periodontal pathogens affecting the intestinal barrier will yield new insights into the significance of periodontal treatment for the prevention and cure of gastrointestinal and systemic diseases.

## Author Contributions

YL and YK made substantial contributions to the conception and design of the study. YL, WH, JW, JM, MZ, XL, JL, and YK participated in drafting the article. YL and YK gave final approval of the version to be submitted and revised versions.

## Funding

Natural Science Foundation of Liaoning Province (20180551232) and Science and Technology Project of Shenyang (F16-102-4-00).

## Conflict of Interest

The authors declare that the research was conducted in the absence of any commercial or financial relationships that could be construed as a potential conflict of interest.

## Publisher’s Note

All claims expressed in this article are solely those of the authors and do not necessarily represent those of their affiliated organizations, or those of the publisher, the editors and the reviewers. Any product that may be evaluated in this article, or claim that may be made by its manufacturer, is not guaranteed or endorsed by the publisher.
